# Identifying Distinct Developmental Patterns of Brain Complexity in Autism: A Cross‐Sectional Cohort Analysis Using the Autism Brain Imaging Data Exchange

**DOI:** 10.1111/pcn.13780

**Published:** 2025-01-11

**Authors:** I‐Jou Chi, Shih‐Jen Tsai, Chun‐Houh Chen, Albert C. Yang

**Affiliations:** ^1^ Institute of Brain Science National Yang Ming Chiao Tung University Taipei Taiwan; ^2^ Department of Psychiatry Taipei Veterans General Hospital Taipei Taiwan; ^3^ Institute of Statistical Science Academia Sinica Taipei Taiwan; ^4^ Department of Medical Research Taipei Veterans General Hospital Taipei Taiwan; ^5^ Digital Medicine and Smart Healthcare Research Center National Yang Ming Chiao Tung University Taipei Taiwan

**Keywords:** autism spectrum disorder, brain complexity, developmental trajectories, functional magnetic resonance imaging, sample entropy

## Abstract

**Aim:**

Autistic traits exhibit neurodiversity with varying behaviors across developmental stages. Brain complexity theory, illustrating the dynamics of neural activity, may elucidate the evolution of autistic traits over time. Our study explored the patterns of brain complexity in autistic individuals from childhood to adulthood.

**Methods:**

We analyzed functional magnetic resonance imaging data from 1087 autistic participants and neurotypical controls aged 6 to 30 years within the ABIDE I (Autism Brain Imaging Data Exchange) data set. Sample entropy was calculated to measure brain complexity among 90 brain regions, utilizing an automated anatomical labeling template for voxel parcellation. Participants were grouped using sliding age windows with partial overlaps. We assessed the average brain complexity of the entire brain and brain regions for both groups across age categories. Cluster analysis was conducted using generalized association plots to identify brain regions with similar developmental complexity trajectories. Finally, the relationship between brain region complexity and autistic traits was examined.

**Results:**

Autistic individuals may tend toward higher whole‐brain complexity during adolescence and lower complexity during childhood and adulthood, indicating possible distinct developmental trajectories. However, these results do not remain after Bonferroni correction. Two clusters of brain regions were identified, each with unique patterns of complexity changes over time. Correlations between brain region complexity, age, and autistic traits were also identified.

**Conclusion:**

The study revealed brain complexity trajectories in autistic individuals, providing insight into the neurodiversity of autism and suggesting that age‐related changes in brain complexity could be a potential neurodevelopmental marker for the dynamic nature of autism.

Autism spectrum disorder, characterized by atypical social skills and ritualistic behaviors, was first identified by Leo Kanner in 1943, who noted a distinct pattern of behavior among children with autism.^1^ Kanner's subsequent observations 30 years later revealed significant variability in the behaviors and outcomes of adults with autism.^2^, ^3^ This variability suggests the importance of considering autism's developmental trajectory as a whole, given that autistic traits change dynamically over a lifespan. A recent review found both stable and changing patterns in autistic traits, pointing to the complexity and diversity in the characteristics of autism.^4^ This diversity poses challenges for diagnosis, treatment, and support,^5^, ^6^ emphasizing the need for further research into the mechanisms underlying changes in autistic traits across different developmental stages.

We have developed a framework to explain the wide range of behavioral traits observed across different spectrums through brain complexity.^7^ This framework is based on the idea that complexity lies between order and randomness, serving as a measure of predictability for a given component within a system of multiple interactions. Complexity is linked to both intrinsic neural activities and external cognitive performances in brain systems, relevant to age‐related neural network activities and organizations,^8^, ^9^ which are crucial for adapting to a constantly changing context.^8^ The complexity theory can be applied from microscopic neural activities^10^ to macroscopic human behaviors.^11^ A complex brain system has inherent interactive features that enable it to effectively navigate daily fluctuations. If a brain system exhibits excessive chaos or monotony, lacking distinct or engaging interaction patterns among its components, it would be considered to have low complexity.^7^, ^12^ Investigating neural system complexity in autism could offer insights into the evolving patterns of autistic traits across different ages.

Previous studies on brain complexity in autism have yielded inconsistent results. Research has identified a significant shift toward randomness in brain activity among children and adults with autism.^13^, ^14^, ^15^ Another study found increased entropy in the superior frontal gyrus but reduced sample entropy in children with autism compared with their typically developing peers, suggesting differences based on the complexity measurements used.^16^ Conversely, some studies reported no significant complexity differences between youths with and without autism.^17^ Additionally, variability within the autism spectrum has been observed, with severe autism linked to more pronounced reductions in brain complexity than mild autism, indicating a potential relationship between the degree of complexity reduction and the severity of autistic traits.^18^ The disparities in these findings could stem from differences in participant age, measurement modalities, complexity evaluation parameters, and the specific brain regions examined. Further research employing consistent variables is necessary to enhance our understanding of brain complexity in autism among different developmental stages.

Based on prior research, autistic traits are known to vary throughout development, with brain complexity potentially serving as an underlying mechanism. This study aimed to leverage complexity theory to trace the developmental trajectory of brain complexity in individuals with autism across various brain regions and life stages, from childhood to adolescence and young adulthood. We also aimed to identify clusters of brain regions showing distinct patterns of developmental trends in brain complexity. Furthermore, we intended to determine the relationship between the trajectory of brain complexity and autistic traits. Our goal is to deepen our understanding of whether brain complexity theory can explain shifts in autistic traits, offering insights that could pave the way for enhanced diagnostic methods, phenotyping, and support for individuals with autism throughout their developmental phases.

## Methods

### Participants

Preprocessed functional magnetic resonance images from 1087 participants with autism and neurotypical controls, aged 6 to 30 years, from the ABIDE I (Autism Brain Imaging Data Exchange) preprocessed data set version I (https://fcon_1000.projects.nitrc.org/indi/abide/)^19^ were analyzed. The ABIDE I data set included participants aged 6 to 64 years. In this study, the neurotypical group referred to typically developing participants; the autistic group included participants diagnosed with autism spectrum disorders according to the DSM‐IV‐TR demographic records across ABIDE I. Participants lacking magnetization‐prepared rapid gradient echo and functional magnetic resonance imaging (fMRI) data or with data of unexpected quality were excluded. Participants in both the neurotypical and autistic groups were further divided into several age groups using a sliding age window protocol.^20^ They were grouped every 5 years, with 4 years overlapping with the previous group, to create smooth developmental trajectories of brain complexity.

### Measuring brain complexity using sample entropy

The value of brain complexity was calculated by sample entropy from the resting‐state fMRI signal of each voxel. Sample entropy is a measure used to assess the complexity of physiological time‐series signals and diagnose diseased states.^21^, ^22^, ^23^ In general, higher sample entropy values indicate a greater degree of randomness or irregularity in the time series, meaning fewer repeating patterns exist in the data. Lower sample entropy values, on the other hand, indicate a greater degree of regularity or periodicity, meaning that there are more repeating patterns in the data.^21^

The average brain complexity in 90 brain regions was measured by the sample entropy of the time series obtained from each gray matter voxel parcellating by the automated anatomical labeling (AAL) template.^24^ Due to various time points among institutions in ABIDE (Table S2), the parameter setting would be crucial for sample entropy. The theoretical recommendation for sample entropy is that the length of the time series has to be larger than 10^m^,^22^ so the choice of m is critical to the statistical validity of the results. We previously published an article on reducing bias in entropy estimates of resting‐state fMRI signals.^25^ In this paper, because the shortest epoch length was 77 (>10^1^ but <10^2^), we chose the parameters of 1, 0.15, and 1 for *m, r*, and scale factor to minimize calculating errors. To track the development of brain complexity, we analyzed the average complexity of the entire brain and brain regions in autistic and healthy participants across different age groups.

### Statistical analysis

This study utilized MATLAB 2021b (The MathWorks, Inc.) and spss version 21.0 (IBM) to analyze descriptive statistics, conduct independent *t* tests, and calculate multiple regression, with the significance level corrected by the Bonferroni correction.^26^ Descriptive statistics were analyzed to understand the characteristics of each age group. To construct the trajectories of brain complexity, we connected the mean values of the whole brain and brain regions in each group among autistic participants and healthy controls separately. Data were tested for distribution by Kolmogorov–Smirnov test.^27^ The Generalized Extreme Studentized Deviate Test^28^ was performed to detect outliers in autism and control groups, analyzed separately for each age group. We calculated the differences between autistic individuals and healthy controls for both the whole brain and specific brain regions in each group.

We then conducted cluster analysis to identify subsets of brain regions with similar trends in brain complexity across age groups. Generalized association plots (GAP), developed by Chen, were used for cluster analysis and result visualization (https://gap.stat.sinica.edu.tw/Software/index.htm). GAP proved particularly useful in understanding the relationships between multiple variables within a data set and uncovering patterns not readily apparent through traditional methods.^29^ GAP represents the associations between variables in an intuitively visual manner, extending the concept of heatmaps to display both individual variable correlations and conditional associations, facilitating the exploration of complex relationships among variables. In our study, GAP helped to cluster brain regions based on their developmental trajectories and visualize the relationships between brain region distribution and complexity development patterns, utilizing GAP software version 0.2.6.69.

We then first used a variance inflation factor (VIF) test to eliminate the effects of collinearity among complexity about brain regions.^30^, ^31^ Then we conducted multiple regression analysis with a 1000 bootstrapping strategy^32^ for 85% of participants to assess the association between brain complexity in survival regions and autistic traits. The complexity values of brain regions and their corresponding scores on autistic‐relevant behavioral scales in ABIDE I were considered as variables of interest. This approach allowed us to quantify the association between these variables by computing the regression coefficient, facilitating a quantitative assessment and comparison of potential association shifts across different age cohorts, thereby contributing to a deeper understanding of the dynamic interrelationships among these variables.

## Results

### Demographic data

A total of 1087 participants were involved in this study, including 519 autistic participants and 568 neurotypical controls. The participants were divided into 21 age groups, ranging from 6 to 10 years old to 26 to 30 years old. The demographics of the total participants are presented in Table 1 (also refer to Table S1 for the demographics of each age group.).

**Table 1:** Participant characteristics

	Autistic (*n* = 519)	TD (*n* = 568)	*P*‐value
Age, mean ± SD/range (years)	15.20 ± 5.25/7–30	15.73 ± 5.59/6.47–30.78	0.12†
Sex, *n*, %			
Men	458 (88.25)	471 (82.92)	<0.001^‡^, ^§^
Women	61 (11.75)	97 (17.08)	
FIQ, mean ± SD	104.50 ± 20.83	107.50 ± 21.94	0.02^†^
Current medicine status			
Yes (%)	22.54	1.58	<0.001^‡^ ^,§^
Handedness (%)			
Right	83.04	91.20	<0.001^‡^, ^§^
Left	12.52	5.99	<0.001^‡^, ^§^
Mix	0.58	0.53	0.31^‡^
Ambiguous	3.85	2.29	0.26^‡^

*Note*: Data are mean ± SD or *n* (%) unless specified otherwise.

Abbreviations: FIQ, full intelligence quotient; TD, typically developing individual.

^†^ Independent *t* test.

^‡^ Chi‐square test.

^§^ Significance level <0.05.

### Trajectories of sample entropy in autistic individuals and neurotypical controls

**Fig. 1:**
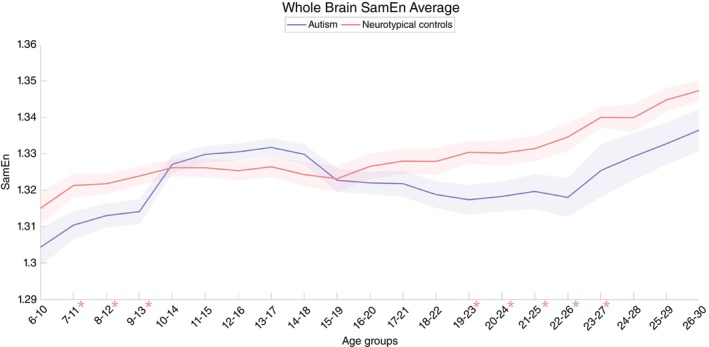
Developmental trajectories of whole brain sample entropy in autism and neurotypical controls. The figure presents the mean sample entropy (SamEn) values of the whole brain, comparing autistic individuals (blue line) with neurotypical controls (red line) across different age groups from 6 to 30 years. The shaded areas indicate the SEM (Standard error of the mean). The *x*‐axis is labeled with age groups, while the *y*‐axis measures the SamEn. Peaks and troughs illustrate the variability in brain complexity at various developmental stages, with autistic individuals showing distinct patterns in comparison to neurotypical controls. *P<0.05. Significant differences were found in the childhood and young adulthood stages, but no statistically significant difference was found after Bonferroni correction (significance level of 0.01).

The sample entropy values in autistic individuals exhibited a modest increase in overall brain complexity during adolescence while showing decreased complexity during childhood and adulthood compared to neurotypical controls (Fig. 1). In whole‐brain mean sample entropy, after removing outliers in each age group of autism and neurotypical controls, significant differences were found in the childhood and young adulthood stages, including the groups aged 7 to 11 years (autism: 1.31 ± 0.05, controls: 1.32 ± 0.04; *P* = 0.04), 8 to 12 years (autism: 1.31 ± 0.04, controls: 1.32 ± 0.04; *P* = 0.04), 9 to 13 years (autism: 1.31 ± 0.05, controls: 1.32 ± 0.04; *P* = 0.02), 19 to 23 years (autism: 1.32 ± 0.03, controls: 1.33 ± 0.03; *P* = 0.01), 20 to 24 years (autism: 1.32 ± 0.03, controls: 1.33 ± 0.03; *P* = 0.03), 21 to 25 years (autism: 1.32 ± 0.03, controls: 1.33 ± 0.03; *P* = 0.04), 22 to 26 years (autism: 1.32 ± 0.03, controls: 1.33 ± 0.03; *P* = 0.01), and 23 to 27 years (autism: 1.33 ± 0.04, controls: 1.34 ± 0.03; *P* = 0.02). However, no statistically significant difference was found after Bonferroni correction. The Bonferroni correction adjusts the *P‐*value by dividing it among the 5‐year repeated age groups, resulting in a significance level of 0.01.

**Fig. 2:**
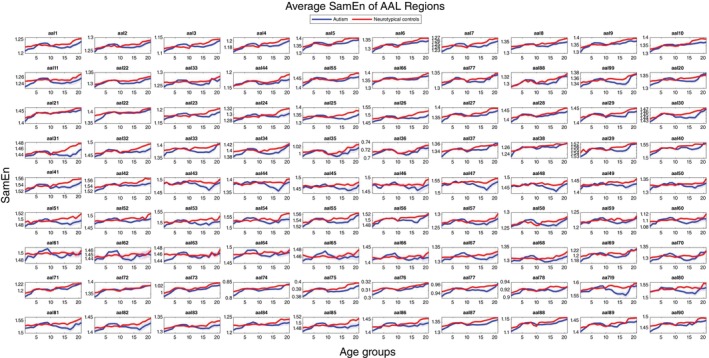
Trajectories of sample entropy in automated anatomical labeling (AAL) brain regions across different age groups between autistic individuals and neurotypical controls. The figure displays the average sample entropy (SamEn) trends for AAL brain regions. Each subplot compares the SamEn values for a specific AAL region between autistic individuals (blue line) and neurotypical controls (red line) across age groups ranging from 6 to 30 years. The shaded areas indicate the SEM. The *x*‐axis is labeled with 21 age groups, with 1 representing the 6‐ to 10‐year‐old group, 2 representing the 7‐ to 11‐year‐old group, and so on.

Furthermore, when examining the 90 AAL brain regions (Table 2) instead of focusing on the whole‐brain trajectories of sample entropy, each brain region displayed its unique trajectory pattern (Fig. 2). Although no significant differences were identified upon correction (the Bonferroni correction adjusts for this *P* value by dividing 5‐year repeated age groups multiplied by 90 brain regions, with a significance level of 0.00012), variations in sample entropy from childhood to adolescence and adulthood were observed, depending on the brain regions. For instance, the left superior frontal gyrus (AAL 3, autism: 1.12 ± 0.03, controls: 1.14 ± 0.03; *P* = 0.002) in the 20‐ to 24‐year‐old group and the right hippocampus (AAL 38, autism: 1.25 ± 0.03, controls: 1.27 ± 0.02; *P* = 0.009) in the 23‐ to 27‐year‐old group exhibited differences. Finally, when plotting the trajectories using the mean values of sample entropy for autistic individuals and neurotypical controls, not every brain region displayed a clear increasing tendency from childhood to adulthood. Some brain regions, such as the fusiform gyrus, exhibited flatter trends in the trajectories of sample entropy for both groups.

**Table 2:** Brain regions in the AAL atlas

AAL	Regions	AAL	Regions
**1/2**	Precental gyrus	**47/48**	Lingual gyrus
**3/4**	Superior frontal gyrus, dorsolateral	**49/50**	Superior occipital gyrus
**5/6**	Superior frontal gyrus, orbital part	**51/52**	Middle occipital gyrus
**7/8**	Middle frontal gyrus	**53/54**	Inferior occipital gyrus
**9/10**	Middle frontal gyrus, orbital part	**55/56**	Fusiform gyrus
**11/12**	Inferior frontal gyrus, opercular part	**57/58**	Postcentral gyrus
**13/14**	Inferior frontal gyrus, triangular part	**59/60**	Superior parietal gyrus
**15/16**	Inferior frontal gyrus, orbital part	**61/62**	Inferior parietal, but supramarginal and angular gyri
**17/18**	Rolandic operculum	**63/64**	Supramarginal gyrus
**19/20**	Supplementary motor area	**65/66**	Angular gyrus
**21/22**	Olfactory cortex	**67/68**	Precuneus
**23/24**	Superior frontal gyrus, medial	**69/70**	Paracentral lobule
**25/26**	Superior frontal gyrus, medial orbital	**71/72**	Caudate nucleus
**27/28**	Gyrus rectus	**73/74**	Lenticular nucleus, putamen
**29/30**	Insula	**75/76**	Lenticular nucleus, pallidum
**31/32**	Anterior cingulate and paracingulate gyri	**77/78**	Thalamus
**33/34**	Median cingulate and paracingulate gyri	**79/80**	Heschl gyrus
**35/36**	Posterior cingulate gyrus	**81/82**	Superior temporal gyrus
**37/38**	Hippocampus	**83/84**	Temporal pole: superior temporal gyrus
**39/40**	Parahippocampal gyrus	**85/86**	Middle temporal gyrus
**41/42**	Amygdala	**87/88**	Temporal pole: middle temporal gyrus
**43/44**	Calcarine fissure and surrounding cortex	**89/90**	Inferior temporal gyrus
**45/46**	Cuneus		

*Note*: Hemisphere location: odd numbers represent left; even numbers represent right.

Abbreviation: AAL, automated anatomical labeling.

### Cluster analysis of the trajectory of brain complexity among 90 AAL brain regions

**Fig. 3:**
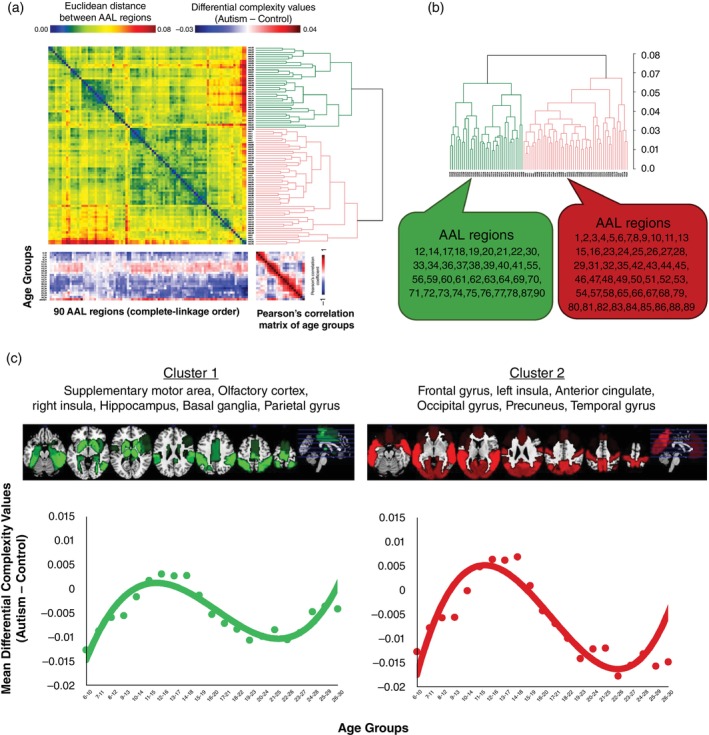
Cluster analysis of trajectories of brain complexity in autism across automated anatomical labeling (AAL) regions. The figure illustrates the outcomes of a cluster analysis using generalized association plots. (a) The heatmap with dendrograms shows the Euclidean distances between the differential complexity values (autism mean sample entropy minus control mean sample entropy) of AAL brain regions in each age group, revealing two main clusters. The square with a rainbow color legend represents the matrix of distances between brain regions. The cool colors including blue and green represent relatively close relationships between two regions; the warm colors including red and yellow represent relatively distant relationships between two regions. The rectangle with a blue‐red color legend represents the differential sample entropy values of autism groups minus control groups of brain regions (columns) across each age group (rows). Blue represents that the sample entropy value in the autism group was less than in the control group; red represents that the sample entropy value in the autism group was greater than in the control group. White represents differential sample entropy values that approach 0, which means there were nearly no differences between the autism group and the control group. Participants older than 31 years were not included in the main analysis of this study because of the relatively small sample size (*n* = 42) and wide age range (31 to 64 years). However, to provide a comprehensive overview of the developmental stages of brain complexity, participants in the older than 31‐year group were still included in the generalized association plot analysis. The square matrix in the bottom right corner represents the Pearson correlation coefficient of the mean sample entropy values between age groups. Blue represents that the relationships between age groups were negative, whereas red represents that the relationships between age groups were positive; white represents correlation coefficient values of 0. (b) AAL brain regions classified within each cluster are listed, with cluster 1 consisting mostly of subcortical areas and cluster 2 including primarily cortical regions. (c) The lower panel depicts the mean differential complexity (autism minus control) for the brain regions within each cluster across age groups, with cluster 1 showing a gentle fluctuation and cluster 2 likely to present a steeper curve, suggesting distinct patterns of complexity change from childhood to adulthood in patients with autism spectrum disorder.

Next, we computed the differential complexity curves for the autistic and control groups within each brain region by subtracting the average complexity trendline of the autistic group from that of the control group. These differential complexity curves captured the variability in complexity differences between the autistic and control groups over different ages for each brain region. Subsequently, we employed the GAP software for analysis to ascertain whether the differential complexity curves among 90 brain regions exhibited clustering characteristics. The visualization of the cluster analysis (Fig. 3a) clearly showed that utilizing the Euclidean distance and complete‐linkage method allowed the categorization of brain regions with similar trends in complexity differences over ages into two distinct clusters (i.e., green for cluster 1 and red for cluster 2).

The dendrogram (Fig. 3b) further elucidated the brain regions encompassed within each cluster. Brain regions within the same cluster indicated shared patterns of complexity change. While most bilateral brain regions were grouped based on similar trend patterns, seven brain regions were separated into clusters, suggesting that they exhibited distinct variation trends compared with their counterparts on the opposite side. These brain regions include the inferior frontal gyrus, opercular part (AAL 11, 12), inferior frontal gyrus, triangular part (AAL 13, 14), insula (AAL 29, 30), posterior cingulate gyrus (AAL 35, 36), amygdala (AAL 41, 42), middle temporal pole (AAL 87, 88), and inferior temporal gyrus (AAL 89, 90).

When projecting the brain regions of individual clusters onto the multi‐sliced images (Fig. 3c), cluster 1 primarily covered the top of the brain and subcortical areas, including the parietal gyrus, paracentral lobule, median cingulate, paracingulate gyri, hippocampus, thalamus, and basal ganglia. Cluster 2 was mainly located in the anterior and posterior brain regions and much of the surface cortex, including the dorsolateral frontal gyrus, temporal poles, angular gyrus, precuneus, occipital lobe, calcarine fissure, and surrounding cortex.

The complexity difference trendlines within brain regions for each cluster were delineated to age groups. This graphical representation highlighted discernible dissimilarities in patterns between the two distinct clusters (Fig. 3c). No significant difference was found (*t* = 1.08, *P* = 0.29) between the trendlines of the two clusters after Student *t* test. However, compared with cluster 2, the rate of complexity differential values among age groups was shown to be more gradual within cluster 1. Commencing similarly from the 6‐ to 10‐year‐old group, the differential values in both clusters converged at approximately −0.017, and both reached their peak differences within the 12‐ to 16‐year‐old group. However, within the 12‐ to 16‐year‐old group, the difference value in cluster 1 amounted to 0.003, whereas in cluster 2 it was 0.007. This divergence imparted a steeper trajectory to the complexity trend of cluster 2 among the child and adolescent cohorts, implying an accelerated elevation in the average complexity of brain regions within cluster 2 of the autistic group during these life stages. On the other hand, while the complexity differential values of both clusters reached their respective minima around similar age groups (cluster 1 in the 21‐ to 25‐year‐old group; cluster 2 in the 22‐ to 26‐year‐old group), the trajectory differed markedly. Cluster 1 declined from 0.003 to approximately −0.01, while cluster 2 showed a reduction from 0.007 to −0.017. This observation suggests that within cluster 2, brain regions of the autistic group underwent a rapid reduction in complexity degrees during the transition from adolescence to adulthood. Further analysis to evaluate the robustness of these clusters is shown in Fig. S1 and Table S3.

### Association of regional brain complexity with autistic traits

The complexity of brain regions was associated with different behavioral traits within the autistic group. After conducting the VIF test, only the complexity of the bilateral posterior cingulate gyrus (AAL 35, 36) and the left amygdala (AAL 41) remained significant. The adjustment for Bonferroni correction for *P*‐values divided five variables in multiple regression, including complexity of AAL 35, 36, and 41, age, and sex; therefore, the significance level is 0.01. The complexity of the bilateral posterior cingulate gyrus was primarily associated with social cognition (AAL 35 beta = 154.36, *P* = 0.01) and social communication (AAL 35 beta = 233.49, *P* = 0.03; AAL 36 beta = −279.78, *P* = 0.05) subscores in the Social Responsiveness Scale (SRS), as well as the scaled score of community daily living skills (AAL 35 beta = −34.98, *P* = 0.04) in the Vineland Adaptive Behavior Scales (VABS).

On the other hand, age was significantly associated with several autistic traits and daily living skills. For example, repetitive restricted behaviors were associated with age in both the Autism Diagnostic Interview‐Revised (ADI‐R) (beta = −0.06, *P* = 0.0076) and the Autism Diagnostic Observation Schedule, Second Edition (ADOS‐2) (beta = −0.03, *P* = 0.0049), communication subscore (beta = 0.03, *P* = 0.0098) in ADOS‐2, and subscore of the abnormality of development evident at or before 36 months (beta = −0.03, *P* = 0.0073) in ADI‐R. In VABS, scores of expressive language (beta = −0.14, *P* = 0.0013), communication (beta = −1.21, *P* = 0.0000005), community daily living skills (beta = −0.16, *P* = 0.0006), daily living skills (beta = −0.74, *P* = 0.0028), interpersonal relationships (beta = −0.15, *P* = 0.0016), and socialization (beta = −0.93, *P* = 0.001) were associated with age. The multiple regression models and results are shown in Table 3.

**Table 3:** Multiple regression models and results

Behavioral scale	$r^{2}$	AAL 35 Complexity (beta value)	AAL 36 Complexity (beta value)	AAL 41 Complexity (beta value)	Age (beta value)	Sex (beta value)	*F*	*P*‐value of the model
ADI‐R								
Restrictive and repetitive behaviors	0.02	5.22	−0.62	−0.19	−0.06^‡^	−0.51	2.22	0.05
Abnormality before 36 months	0.03	−3.85	4.57	−1.65	−0.03^‡^	−0.18	2.84	0.016^†^
ADOS‐2								
Communication	0.01	0.72	0.57	−1.70	0.03^‡^	−0.12	1.56	0.17
Stereotyped behaviors	0.03	0.38	1.24	−3.08	−0.03^‡^	0.24	2.73	0.02^†^
SRS								
Cognition	0.12	154.36^†^	−181.37	−20.81	0.06	−0.80	1.46	0.25
Communication	0.06	233.49^†^	−279.78^†^	−28.95	0.19	−3.95	1.14	0.37
VABS								
Expressive language	0.13	−1.23	−2.94	2.19	−0.14^‡^	−0.40	2.74	0.027^†^
Communication standard score	0.27	13.20	−28.94	14.51	−1.21^‡^	−0.81	5.54	0.0003^†^
Personal daily living skills	0.08	−27.06	42.04	−7.18	−0.10	−1.18	2.22	0.06
Community daily living skills	0.27	−34.98^†^	32.14	−0.72	−0.16^‡^	−0.87	5.67	0.0003^†^
Interpersonal relationships	0.12	−12.12	14.25	11.74	−0.16^‡^	−0.27	2.47	0.043^†^
Sum scores	0.26	−318.57	365.83	12.15	−2.88^‡^	−11.50	5.34	0.0004^†^

*Note*: The behavioral scale values would adopt the scores of ADI‐R, ADOS‐2, SRS, VABS provided from ABIDE I. 
$$\text{Behavioral scale}\;value=r^{2}+b_{\mathrm{AAL}35}X_{\mathrm{AAL}35}+b_{\mathrm{AAL}36}X_{\mathrm{AAL}36}+b_{\mathrm{AAL}41}X_{\mathrm{AAL}41}+b_{\mathrm{age}}\times \mathrm{age}+b_{\mathrm{sex}}\times \mathrm{sex}$$
The *X*_*AAL35*_, *X*_*AAL36*_, and *X*_*AAL41*_ are the complexity values of brain regions that survived from VIF test. The data present in the table, which are coefficient *b* in the formula, are beta values, unless specified otherwise.

Abbreviations: AAL 35, left posterior cingulate gyrus; AAL 36, right posterior cingulate gyrus; AAL 41, left amygdala; ADI‐R, Autism Diagnostic Interview‐Revised; ADOS‐2, Autism Diagnostic Observation Schedule, Second Edition; SRS, Social Responsiveness Scale; VABS, Vineland Adaptive Behavior Scales; VIF, variance inflation factor.

^†^ *P*‐value <0.05.

^‡^ Beta value of the variable's *P*‐value less than the Bonferroni‐corrected significant level 0.01.

## Discussion

This study presents several key findings: (i) The developmental trajectories of autism showed no statistical difference from controls but exhibited slight deviations; (ii) two distinct clusters of brain regions were identified based on complexity differences, highlighting diverse neurodevelopmental patterns within autism; (iii) certain brain regions' complexity, particularly the bilateral posterior cingulate gyrus and the left amygdala, was associated with specific autistic behaviors, although these associations did not remain significant after correction; and (iv) age was significantly correlated with various autistic behaviors and daily living skills, emphasizing the dynamic nature of autism spectrum disorder across development. These findings may shed light on the intricate relationships between brain complexity and autism, revealing both the diverse developmental trajectories of brain complexity and their potential links to autistic traits, highlighting the importance of considering both neurobiological and developmental factors in understanding and supporting individuals with autism.

Sample entropy measures the regularity of resting‐state fMRI signals, with higher values suggesting more random or diverse neural activation patterns.^33^ Ando et al. (2022) proposed that neural complexity is an important index coupling with functional network activities and aging.^9^ In this study, the sample entropy results among neurotypical controls align with previous findings. However, unlike the steady increase in neurotypical controls, autistic individuals' sample entropy peaked during adolescence across the brain, hinting at unique complexity trajectories and neural activity properties. These properties may be influenced by the imbalance of excitatory and inhibitory neurotransmitters in autism.^34^ As the primary inhibitory neurotransmitter γ‐aminobutyric acid (GABA) plays a pivotal role in modulating neural excitability and synchrony,^35^ which could directly influence the hemodynamic responses that fMRI captures. Empirical studies show significant correlations between GABA concentrations and fMRI signals, highlighting how GABA levels affect blood oxygen level–dependent signals and network connectivity.^36^, ^37^ Furthermore, computational models provide a theoretical framework by simulating how local neural dynamics, shaped by GABAergic inhibition, scale up to influence mesoscopic fMRI findings.^38^ Cross‐modal studies combining electrophysiology with fMRI show that local neural activities, including GABAergic processes, are reflected in fMRI signals.^39^ Studies have shown a reduction in GABA synthesis enzymes and receptors in autistic children,^33^, ^40^, ^41^ leading to increased neuronal excitability and reduced complexity attributable to heightened resting‐state fMRI signal intensity.^42^ However, GABA levels rise with age in autism, notably in the left dorsolateral prefrontal cortex of adults, aligning with increasing complexity over time and supporting the theory of changing autistic traits.^43^, ^44^ This suggests that autistic individuals have distinctive brain complexity trajectories, coupling with atypical neural networking caused by GABA imbalance during aging, potentially reflecting underlying neurodevelopmental differences.

Brain complexity is also related to brain structural features and distribution. Brain complexity is crucial in structural‐functional coupling, reflecting human brain activities and whole‐brain information integration.^8^ During early childhood, there was a notable augmentation in the mean whole brain volume within the autistic cohorts in contrast to their typically developing counterparts.^3^, ^45^ Subsequently, the trajectory of whole brain volume in autistic cohorts converged with that of typically developing children during early adolescence. Ultimately, the trajectory of whole brain volume in autistic cohorts demonstrated a continued decrement into adulthood. Notably, autistic cohorts tend to decline in the slope of their whole brain volume trajectory than typically developing individuals.^3^ This phenomenon could be attributed to the deficits in the pruning mechanism in autistic individuals. The pruning deficits in the autistic brain result in a decreased elimination of synapses, potentially contributing to the larger whole brain volume observed before and during adolescence.^46^, ^47^

The observed trajectory of whole brain complexity within the autistic group demonstrates a notable concordance with the identified trajectory of whole brain volume in autistic cohorts, aligning with the proposed spatiotemporal complexity framework in brain activity findings.^8^ During childhood, increasing synapse numbers due to pruning deficits and the concurrent rise in whole brain volume may give rise to two distinctive mechanisms within the autistic population. First, it is plausible that the heightened synaptic density contributes to an elevated oxygen demand, thereby maintaining a relatively stable and consistently high blood oxygen concentration. Second, the failure of synapses to undergo mature differentiation may result in neural operations closely resembling an integrated state,^48^ consequently manifesting as rigid and stereotypical patterns of neural activity during the autistic childhood phase. Hence, these factors collectively contribute to diminished brain complexity in autistic children. As autistic individuals transition into adolescence, the pruning mechanisms stabilize and improve, reaching their zenith during this developmental period. This signifies the maturation and differentiation of synapses and an enhanced efficiency of neural operations approximating a state of segregation.^48^ Consequently, neural activity becomes more flexible and adaptable, leading to a peak in brain complexity among autistic individuals during adolescence.

Complexity patterns in the brain from childhood to adulthood reveal distinct clusters among 90 regions, showing a rise in complexity until mid‐adolescence and a decrease thereafter, with variations in the rate of change. Cluster 1 involved subcortical areas and sensory‐motor regions, while cluster 2 included regions across the cerebral cortex and was mostly relevant to higher‐cognitive areas. Notably, cluster 2 showed higher complexity differences from neurotypical controls during key developmental transitions, indicating diverse developmental trajectories in brain structure and function.^49^, ^50^, ^51^ The results also follow the findings that complexity reflects stronger structure–function coupling in sensory‐motor regions and lower structure–function coupling in higher‐cognitive areas.^8^ As adolescents, autistic individuals may reach complexity levels comparable to or exceeding neurotypical peers, possibly due to compensatory mechanisms.^52^ Yet, transitions into adulthood introduce new challenges, impacting brain morphology and complexity differences.^53^, ^54^ These findings suggest the interplay between brain complexity, structural and functional development, and autistic traits, offering insights into the evolving nature of autism spectrum conditions.

Most bilateral brain regions show similar complexity trajectories, indicating consistent age‐related patterns. Yet, specific areas such as the inferior frontal gyrus, insula, posterior cingulate gyrus, amygdala, and temporal regions fall into different clusters, highlighting varied lateralization effects.^55^ This diversity suggests autism's complex neurodevelopment, where abnormal hemispheric lateralization is linked to language and behavioral traits.^55^, ^56^ Particularly, the left hemisphere, apart from the amygdala and middle temporal pole, shows slower complexity changes, suggesting potential lateralization‐related challenges in autism. This analysis not only reveals lateralization patterns in autism but also aids in understanding the nuanced development of neural activity across different stages, enhancing our grasp of autism's neurological underpinnings.

Differences in complexity between regions should be interpreted based on the context and functions of regions and networks. For example, typically developing controls show an age‐related increase in between‐node connectivity within the default mode network (DMN), a pattern not seen in autistic children.^57^ This phenomenon can also be seen in Fig. 2, where the DMN nodes of the precuneus (AAL 67, 68) and middle temporal gyrus (AAL 85, 86) show that the complexity trajectories in autism do not increase with age as they do in typically developing controls. This decoupling of complexity and aging within the DMN suggests abnormalities in the autistic brain's development, such as neurotransmitter imbalances and pruning deficits, leading to nonlinear patterns.^58^, ^59^ On the other hand, Fig. 2 shows that the DMN nodes of the insula (AAL 29, 30) and posterior cingulate gyrus (AAL 35, 36) exhibit more consistent trajectory patterns between patients with autism and typically developing controls. This may imply that the physiological and biochemical developmental mechanisms in the autistic brain vary among different brain regions, warranting further investigation.

Establishing complexity trajectories can enhance the understanding of autism's developmental patterns. Our analysis revealed correlations between complexity in the bilateral posterior cingulate gyrus, age, and autism‐related behaviors. Sample entropy in this data set is linked to autism's core traits, daily living skills, and social functions, assessed by ADI‐R, ADOS‐2, SRS, and VABS. Notably, higher scores in ADI‐R, ADOS‐2, and SRS typically signal stronger autistic traits and social challenges, whereas higher VABS scores reflect better adaptive and cognitive abilities.^60^, ^61^, ^62^, ^63^, ^64^ Our findings suggest that while the bilateral posterior cingulate gyrus's complexity has some impact, age more significantly influences autism behaviors, highlighting autism's dynamic nature. Furthermore, exploring the developmental trajectories of brain complexity provides valuable insights beyond studies focused on specific age groups in autism. Critical periods, occurring postnatally and ending around 5 to 6 years of age, are times when the brain is highly receptive to environmental stimuli, allowing rapid learning and neural plasticity,^65^, ^66^ crucial for understanding developmental disorders such as autism. Although our study did not include very young children, it is plausible that earlier disruptions during these critical periods influenced our participants' brain complexity and clinical symptoms. Research has shown that disruptions in critical periods can lead to long‐lasting changes in neural circuitry and behavior,^67^ which are relevant to the development and manifestation of autism. Furthermore, sensitive periods could relate more to our participants' age range. Sensitive periods in brain development are prolonged intervals where plasticity progressively changes in response to environmental influences.^65^ Sensitive periods are not irrevocably closed by molecular factors and can be reopened by altering environmental conditions. The formation of different connectivity patterns during sensitive periods is reversible and remains functionally flexible.^68^ Future research should include younger cohorts to examine how critical period disruptions affect autism symptoms over time. Additionally, exploring how brain complexity regulates neural system‐environment interactions during sensitive periods is vital. Complexity varies across brain regions and life stages, showing an increasing role in autism‐related regions over time. These patterns highlight the brain's specialization and evolution into a complex system. Our research suggests that understanding these trajectories could enhance our ability to predict and comprehend the functional outcomes of autistic individuals across developmental stages.

## Limitations

To increase generalizability and focus on biological variability, we treated different data collection protocols as covariates in early feature selection regression, which minimally impacts the overall analysis. However, biases from varying measurement conditions exist; hence, external cohort validation is necessary. Despite limited sex‐related data, the influence of sex on neurobehavioral phenotypes cannot be overlooked.^69^ Other factors such as ecological pressures, genetic variations, and social dynamics also significantly influence behavior and brain complexity.^5^, ^6^ Understanding the complex, multifaceted relationship between brain complexity and behavior requires further research with more variables. Given that complexity and behavior likely follow nonlinear patterns, future analyses should include methodologies that capture nonlinear associations for a comprehensive evaluation. Expanding research to include more longitudinal data sets with neuroimaging and behavioral measures will enhance our understanding of brain complexity trajectories in autism. After Bonferroni correction, brain complexity results between autism and controls lacked statistical significance, requiring cautious interpretation and future validation with alternative methods. The observed link between age and severity may be influenced by our sample formation, especially in ABIDE based on DSM‐IV‐TR, likely resulting in only the most severe cases being included among older participants, who were also fewer in number. Further research with more representative samples and modern diagnostic criteria is needed to validate our observations.

## Conclusions

This study presents the dynamic interplay between brain complexity and autism across various life stages, revealing distinct clusters of brain regions and their association with autistic behaviors. While the findings did not reach statistical significance, the trends observed across different brain regions provide meaningful insights into developmental changes in brain complexity in autism. Furthermore, the research identifies the association of age and brain complexity with autistic traits and living skills, suggesting the necessity of a comprehensive approach that incorporates neurobiological, developmental, and environmental factors. Overall, this study highlights the intricate relationship between brain complexity and autism, delineating variable developmental trajectories to deepen our understanding of autism.

## Disclosure statement

The preprocessed brain images, demographics, and behavioral data from the ABIDE I data set was obtained on the Preprocessed Connectomes Project (PCP) (http://preprocessed-connectomes-project.org/abide/download.html). On behalf of all of the authors, the corresponding author states that there are no conflicts of interest.

## Author contributions

I.J.C. analyzed the data, performed the computational analysis, and wrote the manuscript. A.C.Y. designed the study and obtained the necessary funding resources. S.J.T. gave critical comments and contributed to manuscript writing. All authors discussed the results and approved the manuscript. C.H.C. offered suggestions for the statistical analysis and provided consultation on addressing GAP software.

## Supporting information


**Figure S1.** 4 Calinski‐Harabasz values and number of clusters. (Page 4 in SuppInfo file).

**Table S1.** Demographic characteristics in each age group. (Page 1 in SuppInfo file).
**Table S2.** fMRI parameters of ABIDE institutions. (Page 3 in SuppInfo file).
**Table S3.** Results of Cluster Analysis Evaluations. (Page 6 in SuppInfo file).
