# Supplementary figures and images for: Identifying Distinct Developmental Patterns of Brain Complexity in Autism: A Cross‐Sectional Cohort Analysis Using the Autism Brain Imaging Data Exchange
Source: Psychiatry Clin Neurosci. 2025 Jan 11;79(3):98–107. doi: 10.1111/pcn.13780 (PMC11874071; doi:10.1111/pcn.13780)

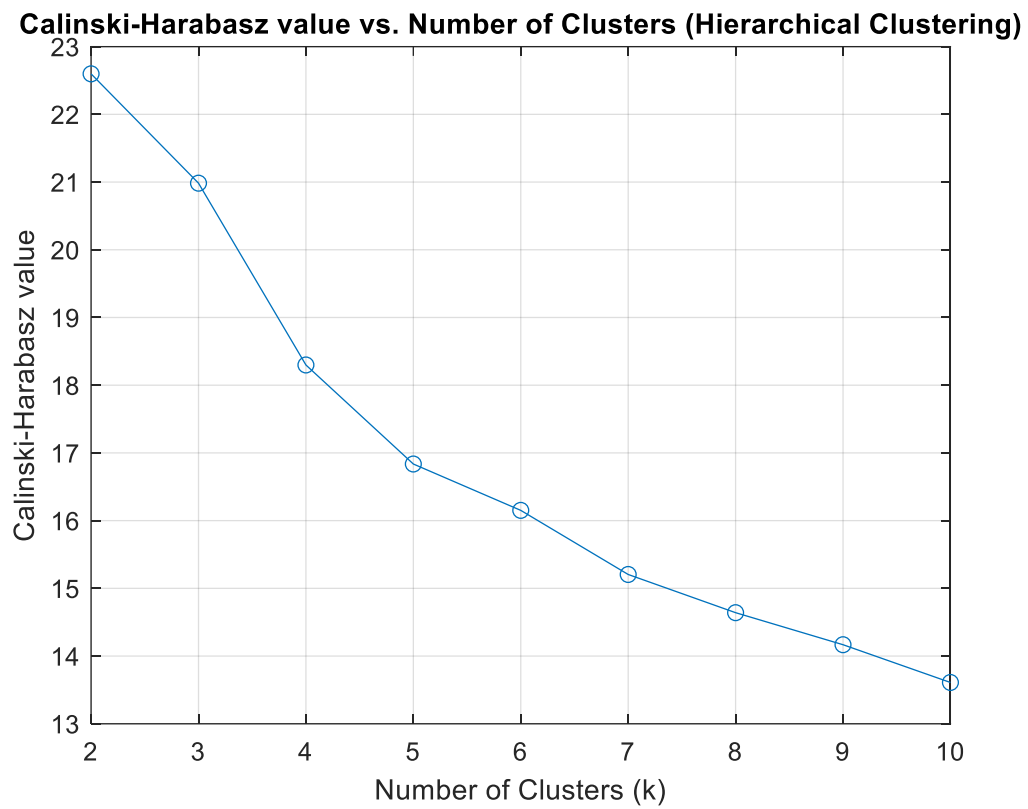

**Figure S1.** Calinski-Harabasz values and number of clusters.

Supplement: Supplementary file 1 — Figure S1. 4 Calinski‐Harabasz values and number of clusters. (Page 4 in SuppInfo file). [file PCN-79-98-s001.pdf]
