# Supplementary material for: Identifying Distinct Developmental Patterns of Brain Complexity in Autism: A Cross‐Sectional Cohort Analysis Using the Autism Brain Imaging Data Exchange
Source: Psychiatry Clin Neurosci. 2025 Jan 11;79(3):98–107. doi: 10.1111/pcn.13780 (PMC11874071; doi:10.1111/pcn.13780)
Supplement: Supplementary file 2 — Table S1. Demographic characteristics in each age group. (Page 1 in SuppInfo file). Table S2. fMRI parameters of ABIDE institutions. (Page 3 in SuppInfo file). Table S3. Results of Cluster Analysis Evaluations. (Page 6 in SuppInfo file). [file PCN-79-98-s002.docx]

**Supplementary Information**

**Table S1.** Demographic characteristics in each age group

| Group  of  age | Autism | | | | TD | | | |
| --- | --- | --- | --- | --- | --- | --- | --- | --- |
|  | Sample size | Sex,  M/F | Age | | Sample size | Sex,  M/F | Age | |
|  |  |  | Mean | SD |  |  | Mean | SD |
| 6 ~ 10 | 103 | 89/14 | 9.35 | 1.09 | 114 | 92/22 | 9.48 | 1.02 |
| 7 ~ 11 | 150 | 128/22 | 10.00 | 1.33 | 149 | 121/28 | 9.99 | 1.22 |
| 8 ~ 12 | 185 | 156/29 | 10.77 | 1.40 | 194 | 161/33 | 10.73 | 1.43 |
| 9 ~ 13 | 192 | 166/26 | 11.60 | 1.35 | 204 | 166/38 | 11.65 | 1.42 |
| 10 ~ 14 | 200 | 172/28 | 12.49 | 1.37 | 220 | 175/45 | 12.61 | 1.43 |
| 11 ~ 15 | 195 | 169/26 | 13.21 | 1.41 | 213 | 168/45 | 13.48 | 1.37 |
| 12 ~ 16 | 184 | 162/22 | 14.32 | 1.49 | 207 | 165/42 | 14.25 | 1.39 |
| 13 ~ 17 | 161 | 145/16 | 15.29 | 1.41 | 183 | 143/40 | 15.21 | 1.37 |
| 14 ~ 18 | 140 | 127/13 | 16.04 | 1.35 | 156 | 131/25 | 15.96 | 1.36 |
| 15 ~ 19 | 116 | 107/9 | 17.03 | 1.28 | 119 | 105/14 | 16.97 | 1.33 |
| 16 ~ 20 | 106 | 97/9 | 17.97 | 1.42 | 100 | 86/14 | 17.97 | 1.42 |
| 17 ~ 21 | 83 | 78/5 | 19.12 | 1.44 | 85 | 71/14 | 19.06 | 1.43 |
| 18 ~ 22 | 77 | 71/6 | 20.36 | 1.52 | 76 | 63/13 | 20.40 | 1.41 |
| 19 ~ 23 | 68 | 62/6 | 21.01 | 1.40 | 76 | 65/11 | 21.36 | 1.37 |
| 20 ~ 24 | 59 | 53/6 | 21.87 | 1.34 | 70 | 60/10 | 22.02 | 1.38 |
| 21 ~ 25 | 47 | 42/5 | 22.82 | 1.37 | 63 | 57/6 | 22.84 | 1.39 |
| 22 ~ 26 | 38 | 33/5 | 23.72 | 1.46 | 55 | 52/3 | 23.75 | 1.44 |
| 23 ~ 27 | 27 | 24/3 | 25.30 | 1.52 | 50 | 48/2 | 25.19 | 1.63 |
| 24 ~ 28 | 27 | 24/3 | 26.22 | 1.55 | 42 | 38/4 | 26.40 | 1.39 |
| 25 ~ 29 | 27 | 25/2 | 27.52 | 1.39 | 43 | 37/6 | 27.23 | 1.41 |
| 26 ~ 30 | 26 | 25/1 | 28.29 | 1.25 | 42 | 35/7 | 28.14 | 1.40 |
| Above 31 | 42 | 36/6 | 38.16 | 8.16 | 36 | 33/3 | 37.66 | 5.71 |

Abbreviations: TD = typically developing; SD = standard deviation; M = male; F = female.

**Table S2.** The fMRI parameters of ABIDE institutions

| **Institution code** | **Institution ID** | **Participant number**  **(age range)** | **Time points** | **Repetition time (ms)** | **Slices** |
| --- | --- | --- | --- | --- | --- |
| 1 | CALTECH | 38 (17.0-56.2 years) | 145 | 2000 | 34 |
| 2 | CMU | 27 (19-40 years) | 315 | 2000 | 28 |
| 3 | KKI | 55 (8.0-12.8 years) | 151 | 2000 | 21 |
| 4 | LEUVEN_1 | 29 (18-32 years) | 245 | 2500 | 47 |
| 5 | LEUVEN_2 | 35 (12.1-16.9 years) | 245 | 1667 | 32 |
| 6 | MAX_MUN | 57 (7-58 years) | 195 | 1667 | 32 |
| 7 | NYU | 184 (6.5-39.1 years) | 175 | 3000 | 40 |
| 8 | OHSU | 28 (8.0-15.2 years) | 77 | 2000 | 33 |
| 9 | OLIN | 36 (10-24 years) | 205 | 2500 | 36 |
| 10 | PITT | 57 (9.3-35.2 years) | 195 | 2700 | 29 |
| 11 | SBL | 30 (20-64 years) | 195 | 1500 | 29 |
| 12 | SDSU | 36 (8.7-17.2 years) | 175 | 2200 | 38 |
| 13 | STANFORD | 40 (7.5-12.9 years) | 235 | 2000 | 42 |
| 14 | TRINITY | 49 (12.0-25.9 years) | 145 | 2000 | 29 |
| 15 | UCLA_1 | 73 (8.4-17.9 years) | 115 | 2000 | 38 |
| 16 | UCLA_2 | 26 (9.8-16.5 years) | 115 | 3000 | 34 |
| 17 | UM_1 | 110 (8.2-19.2 years) | 295 | 3000 | 34 |
| 18 | UM_2 | 35 (12.8-28.8 years) | 295 | 2000 | 40 |
| 19 | USM | 101 (8.8-50.2 years) | 235 | 2000 | 40 |
| 20 | YALE | 56 (7.0-17.8 years) | 195 | 2000 | 40 |

*The time points were less than the raw data length due to the ABIDE fMRI data preprocessing, which included removing the first five time points.

**The cluster analysis evaluations**

Clustering analysis plays a critical role in understanding complex data structures, allowing for the identification of underlying patterns or groupings within datasets. The stability and consistency of clustering results are essential for ensuring the validity and reliability of the identified clusters. Evaluating the robustness of these clusters would be a crucial step.

First, to determine the optimal number of clusters for the entire dataset, we used the Calinski-Harabasz Index. This metric assesses clustering quality by evaluating the ratio between-cluster dispersion to within-cluster dispersion, where higher values indicate more distinct clusters^1^. Compared to other cluster evaluation measures like the silhouette score, the Calinski-Harabasz Index is computationally efficient, applicable to any clustering algorithm and number of clusters, and less sensitive to noise and outliers^2^. Our analysis showed that two clusters were optimal, as the Calinski-Harabasz Index peaked at this point, indicating the most significant separation between the clusters (Figure S1).

**Figure S1**. Calinski-Harabasz values and number of clusters.

Next, for the internal evaluation of the clustering results, we employed the cophenetic correlation coefficient to assess the similarity between the clustering distances in the dendrogram and the actual distances in the original data^3^. The cophenetic correlation coefficient was 0.49, indicating that the dendrogram moderately reflects the structure of the original data.

After confirming that the optimal number of clusters for the entire dataset is two and that the dendrogram moderately reflects the original data distances, we further examined the consistency of the clustering results using a random sampling without replacement approach. For each iteration, we randomly sampled 70%, 80%, and 90% of the cases from the original dataset, performing the same clustering analysis and evaluation, repeated 100 times. On average, based on 70% sampling, the best number of clusters remained two (with 91 out of 100 iterations showing two clusters, 5 showing three clusters, and 2 showing four and five clusters separately). The average cophenetic correlation coefficient across the 100 iterations was 0.54. In summary, the results of the repeated sampling confirm that the clustering method consistently identifies two clusters as the optimal grouping for this dataset, moderately reflecting the original data distances and demonstrating stable clustering results.

In addition to validating the dendrogram and original data as internal evaluations, we also evaluated by comparing the dendrogram linkages and cluster contents from each randomly sampled grouping to the clustering results of the entire dataset as external evaluations.

Firstly, since the two dendrograms are generated from their respective linkage matrices, we directly compared the similarity of the linkage matrices using the Frobenius norm^4^. The Frobenius norm measures the square root of the sum of the squares of all elements in a matrix, with smaller values indicating closer similarity between the matrices^4,5^. The average Frobenius value between the dendrograms generated from 100 random subsampling based on 90% samples and the original dendrogram was 0.24, suggesting that the matrices from the random samples are quite close to the original matrix.

Secondly, we used the Jaccard Index to compare the similarity between the cluster elements (brain regions) from each random sample and the original clustering results. The Jaccard Index measures the similarity between two sets by dividing the size of the intersection by the size of the union, with values closer to 1 indicating higher similarity^6,7^. The average Jaccard Index between the clusters generated from 100 random subsampling and the original clusters was 0.71, indicating a high similarity level of cluster elements.

We also performed a permutation test by holding the age group order fixed and randomly shuffling the sequence of individual brain regions within each age group. This process was repeated 100 times to observe whether the clustering results and evaluation metrics would no longer effectively reflect the original data after disrupting the brain region structure.

For the internal evaluation, the permutation test resulted in an average optimal number of clusters of 2.21, with a mean cophenetic correlation coefficient of 0.37, indicating that the clustering of the shuffled data could not effectively reflect the original structure and with a more varied range of optimal number of clusters.

For the external evaluation, the Frobenius norm was 1.14, showing that the matrices generated from the shuffled data were much farther from the original matrix than those from the unshuffled data. The Jaccard Index was 0.42, meaning that after shuffling, the cluster elements only had 42% similarity to the original cluster elements.

All the evaluation results are presented in the following table S3.

**Table S3.** Results of Cluster Analysis Evaluations

|  | Internal evaluations | | External evaluations | |
| --- | --- | --- | --- | --- |
|  | the optimal number of clusters | Cophenetic correlation coefficient | Frobenius norm | Jaccard Index |
| Original data | 2 | 0.49 | - | - |
| **Random sampling without replacement** | | | | |
| Randomly sample 70% from original data and average 100 iterations. | 2.11 | 0.54 | 0.57 | 0.59 |
| Randomly sample 80% from original data and average 100 iterations. | 2.08 | 0.53 | 0.38 | 0.60 |
| Randomly sample 90% from original data and average 100 iterations. | 2.03 | 0.51 | 0.24 | 0.71 |
| **Permutation** | | | | |
| Holding the age group order fixed, shuffle individual brain region sequences within each age group 100 times. | 2.21 | 0.37 | 1.14 | 0.42 |

From the results above, we aim to demonstrate the clustering stability by showing that each random sampling produces results similar to the original data, such as consistently identifying two optimal clusters and a mean Cophenetic correlation coefficient of around 0.5. External evaluations using the Frobenius norm and Jaccard Index would illustrate that the distance between the sampled Linkage matrix and the original data matrix is short, and the elements within clusters remain similar. The same metrics would also be applied to permuted data, showing that the clustering structure cannot be replicated, providing counterevidence.

Evaluating our clusters through internal measures, such as the Cophenetic correlation coefficient, helps assess how well the hierarchical clustering represents the data. External metrics like the Frobenius norm and Jaccard Index provide insight into the similarity between different clustering results. They offer a quantitative assessment of how closely they align with the original data. By ensuring stability across repeated sampling and demonstrating divergence when using permuted data, we can confirm the reliability of the clustering structure, thereby strengthening the overall analysis.

**References**

1. Caliński T, Harabasz J. A dendrite method for cluster analysis. *Commun. Stat.* 1974; 3: 1–27.

2. Khan IK, Daud HB, Zainuddin NB, Sokkalingam R, Farooq M, Baig ME, et al. Determining the optimal number of clusters by Enhanced Gap Statistic in K-mean algorithm. *Egypt. Inform. J.* 2024; 27: 100504.

3. Rohlf FJ, Fisher DR. Tests for Hierarchical Structure in Random Data Sets. *Syst. Biol.* 1968; 17: 407–12.

4. Meurant G (ed.). Introductory Material. **In**: Studies in Mathematics and Its Applications. Elsevier; 1999 [Cited 2024 Sep 29]. p. 1–68. [Cited 2024 Sep 29] Available from https://www.sciencedirect.com/science/article/pii/S0168202499800022

5. Weisstein EW. Frobenius Norm. [Cited 2024 Sep 29] Available from https://mathworld.wolfram.com/FrobeniusNorm.html

6. Jaccard P. Etude de la distribution florale dans une portion des Alpes et du Jura. *Bull. Soc. Vaudoise Sci. Nat.* 1901; 37: 547–79.

7. Jaccard P. The Distribution of The Flora in The Alpine Zone. *New Phytol.* 1912; 11: 37–50.
